# Human Atrial Cell Models to Analyse Haemodialysis-Related Effects on Cardiac Electrophysiology: Work in Progress

**DOI:** 10.1155/2014/291598

**Published:** 2014-12-23

**Authors:** Elisa Passini, Simonetta Genovesi, Stefano Severi

**Affiliations:** ^1^Computational Physiopathology Unit, Department of Electrical, Electronic, and Information Engineering, University of Bologna, Via Venezia 52, 47521 Cesena, Italy; ^2^Department of Health Sciences, University of Milano Bicocca, Via Cadore 48, 20900 Monza, Italy

## Abstract

During haemodialysis (HD) sessions, patients undergo alterations in the extracellular environment, mostly concerning plasma electrolyte concentrations, pH, and volume, together with a modification of sympathovagal balance. All these changes affect cardiac electrophysiology, possibly leading to an increased arrhythmic risk. Computational modeling may help to investigate the impact of HD-related changes on atrial electrophysiology. However, many different human atrial action potential (AP) models are currently available, all validated only with the standard electrolyte concentrations used in experiments. Therefore, they may respond in different ways to the same environmental changes. After an overview on how the computational approach has been used in the past to investigate the effect of HD therapy on cardiac electrophysiology, the aim of this work has been to assess the current state of the art in human atrial AP models, with respect to the HD context. All the published human atrial AP models have been considered and tested for electrolytes, volume changes, and different acetylcholine concentrations. Most of them proved to be reliable for single modifications, but all of them showed some drawbacks. Therefore, there is room for a new human atrial AP model, hopefully able to physiologically reproduce all the HD-related effects. At the moment, work is still in progress in this specific field.

## 1. Introduction

In the last fifteen years, the increasing interest towards atrial electrophysiology and atrial fibrillation (AF), together with a greater availability of experimental data, led to remarkable developments in human atrial action potential (AP) models [1–6]. As a matter of fact, cardiac computational modeling constitutes an efficient tool to investigate the ionic mechanisms involved at cell level and has already been used in a variety of clinical contexts, linking patient manifestations to the underlying electrophysiological mechanisms, thus providing useful insights into different atrial pathologies, including AF, especially whenever experimental measurements were lacking or unavailable [6–15].

Haemodialysis (HD) therapy represents a unique model to test* in vivo*, in human, the effects of sudden changes in plasma ionic concentrations and blood volume: in a few hours, patients undergo significant plasma electrolytes variations, together with a significant decrease in extracellular volume. In particular, the HD session causes removal of excess Na^+^ and water, the extent of which depends on the interdialytic weight gain of the patient. Plasmatic K^+^ concentration increases during the interdialytic interval, so that during all HD sessions its level must decrease, while Ca^2+^ variations might change depending on the dialysate Ca^2+^ concentration and its relationship with pre-HD plasma Ca^2+^ levels. [16, 17]. These processes often lead to an increased arrhythmic risk for the patient, both during HD and in the hours following the therapy. Indeed, the incidence of AF in end-stage renal disease patients is high: reported rates vary between 7% and 27% [18, 19], and HD session may promote AF onset [20, 21].

The aim of this paper is first to briefly review the literature concerning applications of the computational approach to the study of the impact of HD therapy on cardiac electrophysiology and then to compare all the currently available human atrial AP models, focusing on their ability to reproduce the electrophysiological changes typically induced by HD sessions, that is, plasma electrolytes and blood volume variations.

The 6 published human atrial models have been considered: Courtemanche et al. [1], Nygren et al. [2], Maleckar et al. [3], Koivumäki et al. [4], Grandi et al. [5], and Colman et al. [6]. All models have been tested for different concentrations of extracellular electrolytes (Na^+^, Ca^2+^, and K^+^) and for cell volume changes. A set of AP and Ca^2+^-transient (CaT) biomarkers has been considered to compare simulation results, for example, AP duration (APD), resting membrane potential (RMP), effective refractory period (ERP), and CaT duration (CaTD).

In addition, since a modification of the sympathovagal balance in favour of vagal activity may occur during HD sessions in patients showing intradialytic AF episodes [20], the acetylcholine-activated K^+^ current (*I*_KACh_) has been added to all models, and the effect of different acetylcholine concentrations has been considered as well.

## 2. Cardiac Cell Modeling and Haemodialysis

Computational models of cardiac AP have already been applied several times to assess the acute effects of HD therapy on cardiomyocyte electrophysiology.

The first attempt in this context was the computational analysis of the heart rate changes during HD [22–24]. Since a reliable model of human sinoatrial node (SAN) AP was lacking (as it is still today), these studies were based on a model of rabbit SAN AP, considering the DiFrancesco-Noble model [25, 26], as modified by Dokos et al. [27]. Simulation results pointed out that changes of blood K^+^, Ca^2+^, and pH produce large heart rate variations, showing how electrolyte and pH changes within physiological range may have a remarkable impact on the pace-making rhythm, independently of the autonomic outflow.

The computational approach has been also used to analyse how Ca^2+^ and K^+^ changes during HD can alter ventricular repolarization and therefore AP duration [28]. In this work, a model of human ventricular AP was considered [29] and model predictions on AP prolongation were validated against a wide range of experimental data, that is, QT interval prolongation recorded during HD sessions. Simulation results pointed out how computational modeling of ventricular AP may be useful to quantitatively predict the complex dependence of AP duration on simultaneous changes in both Ca^2+^ and K^+^. From this study, a model-based clinical indication was inferred: Ca^2+^ content in the dialysis bath should be designed in order to prevent a critical reduction of serum Ca^2+^, especially in HD sessions with a risk of end-HD hypokalaemia.

The same approach has been applied to atrial electrophysiology: a computational model of human atrial AP has been used to confirm that the intradialytic reduction of plasma K^+^ level is associated with P-wave prolongation [30]. When comparing the simulated atrial APs at the beginning and at the end of multiple HD sessions, imposing in the model the extracellular electrolyte concentrations and heart rate equal to the experimental values measured* in vivo*, simulation results showed an increase in the time needed for depolarisation and a reduction of the effective refractory period (ERP), both occurring during HD. These two phenomena, in presence of a trigger, that is, repeated premature atrial impulses, frequently induced by a HD session, might form the electrical substrate for intradialytic AF episodes onset. Consistent results were also obtained when performing the same analysis in a multiscale model of the human atrium and considering a simulated ECG [31].

More recently, we applied computational modelling of atrial cellular electrophysiology to the individual case of a patient in which HD regularly induced paroxysmal AF [20]. Simulation results provided evidence of a slower depolarization and a shortened refractory period in pre-AF versus pre-HD conditions, and these effects were enhanced when adding acetylcholine effect in simulation. Starting from these findings, the possible mechanisms leading to intradialytic AF onset were reviewed and reinterpreted. Notably, in a subsequent study, Buiten et al. [21], using the implantable cardioverter defibrillator remote monitoring function, showed that HD is a trigger for AF episodes. In particular, they showed that a lower concentration of K^+^ in the dialysis bath is associated with a higher probability of AF episodes, as predicted by our model-based simulation results.

It is worth noting that, in all these studies, model inputs were set using experimentally measured quantities, that is, plasma electrolyte concentrations and heart rate. However, the actual* in vivo* extracellular fluid is the interstitial fluid, rather than the blood. Therefore, it could be questioned whether the plasma electrolyte concentrations are a reliable estimate of the interstitial ones, even if this is usually accepted. Indeed, the distribution of free ions between vascular and interstitial compartments has been reported to agree with Donnan theory, which predicts a theoretical ratio between interstitial and plasma concentrations very close to 1 [32].

## 3. Atrial Cell Modeling: Materials and Methods

### 3.1. Computational Models of Human Atrial AP

Starting from the first two human atrial cell models (Courtemanche et al. [1]; Nygren et al. [2]), both published in 1998, four more have been released in the last few years (Maleckar et al. 2009 [3]; Koivumäki et al. 2011 [4]; Grandi et al. 2011 [5]; Colman et al. 2013 [6]). Hereafter, the six models will be referred to using the initial letter of the first and last authors (i.e., CN, NG, MT, KT, GB, and CZ, resp.). All models consist of a set of ordinary differential equations, each one representing a specific dynamic process occurring in the cell, and the number of equations is related to their complexity: the first models are very simple compared to the most recent ones, where a more detailed description of Ca^2+^ handling and cell compartments is included (see Table 1). Moreover, the different parameters and ionic current formulations lead to distinct AP morphologies and properties, for example, AP duration (APD), and CaT duration (CaTD).

Since 1998 several papers comparing atrial model performances have been published, mainly concerning CN and NG models, which for many years have been the only ones available [33–39]. The two most recent reviews [38, 39] compared all models except CZ, considering simulations from single cell to whole heart and including both physiological and pathological conditions, thus assessing the current state of the art in atrial computational modeling. Therefore, the comparison of the peculiar properties of these atrial models exceeds the purpose of this work, which rather aims to investigate the acute effects of HD therapy on atrial electrophysiology.

**Figure 1:**
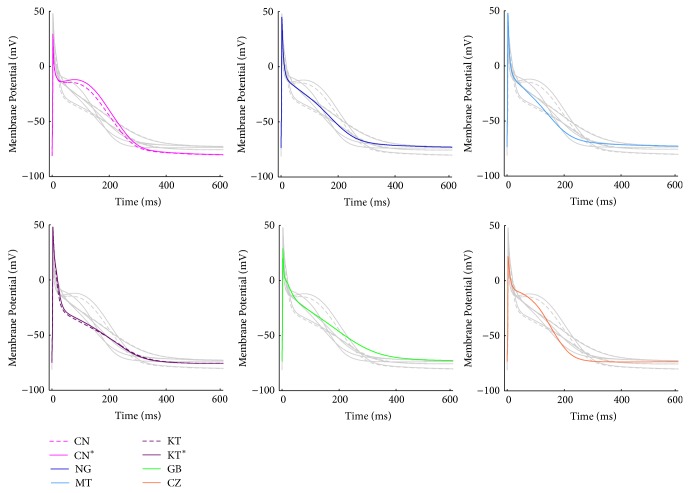
Simulated AP traces for all the considered atrial models: each panel shows a specific model AP with its reference colour; all the other model traces have been added in grey, to facilitate the comparison.

**Figure 2:**
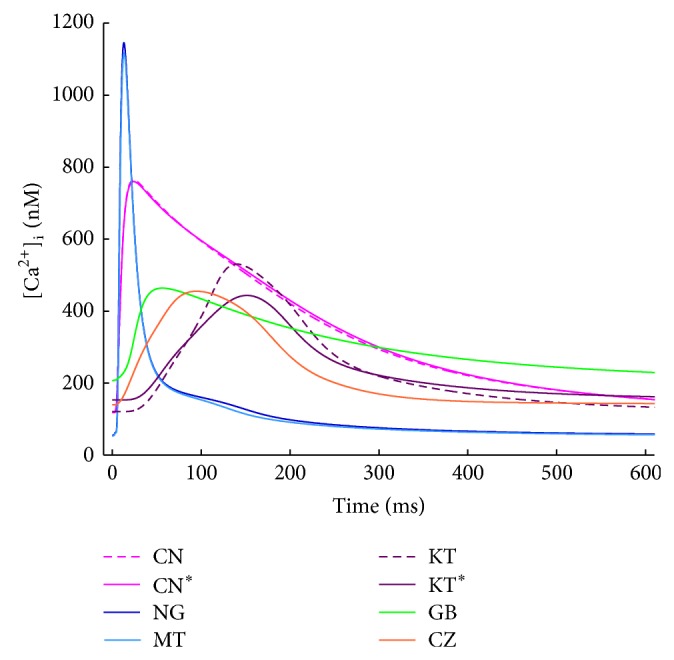
CaT traces for all the considered atrial models: the notable differences in timing and amplitude are related to the corresponding different formulations of Ca^2+^ release from the SR.

The CN and NG models are almost based on the same human atrial data, and they share most of the transmembrane ionic current formulations: however, CN is developed from the guinea pig ventricular model by Luo and Rudy [40], while NG is developed from the atrial rabbit model by Lindblad et al. [41]. The main differences between the two models are related to Ca^2+^-handling and the CaT is much shorter and with a larger amplitude in NG. As a result, their AP shapes are quite different: a spike-and-dome AP for CN and a more triangular one for NG (see Figure 1, pink and blue traces). The MT and KT models are subsequent extensions of NG: the main changes for MT are new formulations for the transient outward (*I*_to_) and ultra-rapid delayed rectifier (*I*_Kur_) currents, while the KT gives a much more detailed description of Ca^2+^-handling, especially concerning Ca^2+^ release. The sarcoplasmic reticulum (SR) is divided into 4 different compartments, including also a spatial dimension: as a result, the CaT is slower compared to the previous model ones, but its duration is increased (see Figure 2, purple trace). The GB model has been developed from the ventricular model published by the same group [42]: most of the ionic current formulations have been preserved and adapted to experimental data acquired in human atrial isolated cardiomyocyte. The AP is quite triangular shaped (see Figure 1, green line), and the Ca^2+^ handling is mostly derived from the rabbit ventricular model by Shannon et al. [43], again adapted to human atrial data. It is worth noting that in this model the intracellular K^+^ concentration is kept constant.

The CZ model is the most recent one: it is based on CN, from which he inherited all the ionic current formulations, except for *I*_to_ and *I*_Kur_, which come from MT. Furthermore, the Ca^2+^ handling has been modified using a structure for the SR similar to the one used in KT, together with the corresponding formulation for Ca^2+^ release and pumps. Conductance has been slightly tuned, to preserve consistency with the original CN model.

In addition to the models listed above, a different version of the CN model has been considered (from now on referred to as CN^*^), slightly modified in order to improve its long term stability [44, 45]. This CN^*^ model has been recently used to investigate the specific case study of a HD patient which presented recurrent intradialytic AF, as described above [20].

Moreover, the KT model has been recently modified by the same authors, improving model prediction in chronic AF [14]. The changes involved mostly L-type Ca^2+^ current (*I*_CaL_) formulation and this new version of the model (from now on referred to as KT^*^) has been considered as well.

Finally, since this study is mainly focused on extracellular electrolyte changes, the known dependence on extracellular K^+^ for both the inward and delayed rectifier K^+^ currents has been added to the atrial models, when not already included [46–49].

Hereafter, each model will be identified by a specific colour (CN/CN^*^, pink; NG, blue; MT, cyan; KT/KT^*^, purple; GB, green; CZ, red) and simulation results for CN and KT will be shown only when a different behaviour with respect to their updated versions (CN^*^ and KT^*^) is found.

Simulated APs and CaTs for all the considered models are shown in Figures 1 and 2, respectively, to allow a quick visual comparison of their main properties. AP and CaT shapes are very different in each model. As far as the CaTs are concerned, experimental values of diastolic intracellular Ca^2+^ concentration reported in literature span from 120 to 230 nM [50–52]: CN, KT, GB, and CZ have values in this range, whereas NG and MT compute lower diastolic concentrations. Measured CaT amplitudes range from 265 to 345 nM [50, 51] and this is best reproduced by CZ and KT. GB produces a slightly smaller CaT while CN, NG, and MT show much higher amplitudes. In addition, the CaT has been reported to decay with a time constant of about 200 ms or even slower [51, 53]: such a slow decay is well reproduced by GB and CN only, while in all the other models it is much faster.

Model differential equations have been implemented in Matlab (Mathworks Inc.) and a variable order solver has been used to solve them (ode 15 s [54]). Pacing was simulated by a current pulse train (pulses of 3 ms, 1 Hz), maintained for 150 s, in order to allow all the models to reach a proper steady state, that is, intracellular concentrations (Na^+^, Ca^2+^, and K^+^) stable over time. Stimulus current (*I*_stim_) amplitude was set to twice the AP threshold for all models, as previously done in [39] (see Table 1). When using this stimulus, however, the GB model produces an AP which is quite different from the one published in the original GB paper: indeed, some of the biomarkers, for example, AP amplitude and upstroke velocity, are highly stimulus-dependent in this model. Therefore, all simulations with the GB model have been done using the stimulus amplitude needed to preserve the original AP characteristics, which is about 6 times the AP threshold and more close as current density to the ones used for the other models. A summary of the considered models and their main properties is reported in Table 1.

### 3.2. Simulation of the HD-Induced Effects

In order to investigate the reliability of each atrial model in reproducing the effects of extracellular electrolyte changes possibly occurring during a regular HD session, we performed a sensitivity analysis by varying the extracellular concentrations ([Na^+^]_o_, [Ca^2+^]_o_, and [K^+^]_o_) around the original model values (130–140 mM, 1.8 mM, and 5.4 mM, resp.). The explored ranges have been set according to the measured values reported in literature for HD patients, extending them to include also possible outliers, as described in Section 4.

At the beginning of the HD session the patient is overhydrated: for this reason, 2-3 litres (or even more) of water is removed from his blood during the treatment. Such a removal is compensated by water refilling from the interstitial fluid and eventually from the intracellular compartment. How fluid accumulation during the interdialytic period and fluid removal during the HD session reflect into variations of intracellular volumes is actually not known in quantitative terms. Therefore, we investigate the effects of a quite large range (±20%) of volume changes.

Finally, to explore the effect of vagal stimulation, we added the acetylcholine-activated K^+^ current (*I*_KACh_) to all the models, according to the formulation suggested in [5] and considering the changes induced by 0–15 nM of acetylcholine (ACh).

### 3.3. AP and CaT Biomarkers

We identified different AP biomarkers in order to quantitatively compare simulation results, choosing in particular the ones already used in previous simulation works either to compare the different atrial AP models [39] or to evaluate the effects induced by electrolyte variations on atrial electrophysiology [30]: action potential duration (APD) was measured as the interval between the AP upstroke and the 90% of repolarization (APD_90_); resting membrane potential (RMP) was measured at the end of diastole; the AP upstroke duration (AP_ud_) was defined as the time needed by membrane voltage to reach 0 mV, starting from the beginning of the pacing pulse [20, 30]; AP amplitude (AP_amp_) was measured as the difference between the AP peak and RMP; maximum upstroke velocity (*dV*/*dt*_MAX_) was computed as the maximum slope during the AP upstroke; the effective refractory period (ERP) was measured by simulating a S_1_-S_2_ protocol: it has been defined as the longest S_1_-S_2_ interval which failed to elicit a S_2_ AP of amplitude >80% of the preceding S_1_ AP [55].

**Figure 3:**
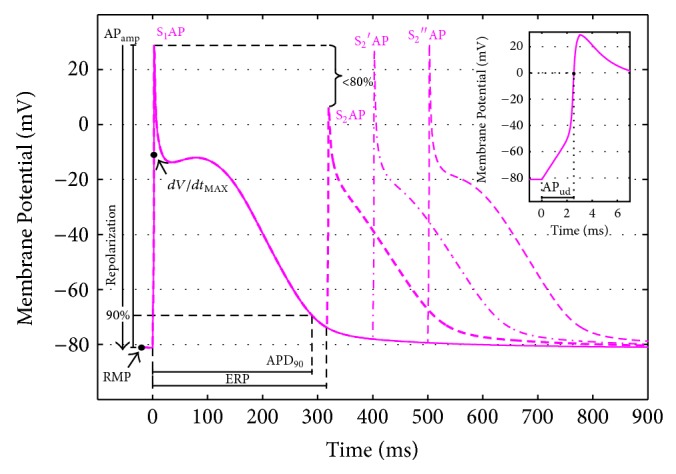
AP biomarkers considered to compare simulation results: AP duration at 90% of repolarization (APD_90_); resting membrane potential (RMP); AP upstroke duration (AP_ud_); AP amplitude (AP_amp_); maximum upstroke velocity (*dV*/*dt*_MAX_); effective refractory period (ERP), computed using a S_1_-S_2_ protocol and considering the longest S_1_-S_2_ interval which failed to elicit a S_2_ AP of amplitude > 80% of the corresponding S_1_ AP [55].

A summary of all the considered AP biomarkers is shown in Figure 3, considering the CN^*^ model AP as an example.

In addition, some Ca^2+^ transient (CaT) biomarkers have been considered as well, that is, CaT duration (CaTD), measured at 90% of CaT decay (CaTD_90_), the time needed to reach the CaT peak, starting from the beginning of the stimulus (CaT_ttp_), and the CaT amplitude (CaT_amp_). Finally, intracellular concentrations ([Ca^2+^]_*i*_, [Na^+^]_*i*_, and [K^+^]_*i*_) have been monitored in all simulations.

## 4. Atrial Cell Modeling: Effects of HD-Related Changes

### 4.1. Potassium Variations

In all the considered models the extracellular K^+^ concentration ([K^+^]_o_) is set to the standard value used in the perfusion bath during V-clamp experiments; that is, 5.4 mM. K^+^ increases during the interdialytic interval and is removed during the HD session: therefore, HD patients often show hyperkalaemia at the beginning of the therapy and hypokalaemia at the end. In order to explore both clinical conditions, we considered the range 3–9 mM. The lower [K^+^]_o_ value (3 mM) has been set considering the experimental post-HD measurements available in literature (e.g., 3.9 ± 0.4 [30], 3.6 ± 0.6 mM [56]). The upper [K^+^]_o_ value (9 mM) is actually a bit high compared to the pre-HD measurements available (e.g., 4.9 ± 0.5 mM in [30], 5.3 ± 0.9 mM in [56]) but we extended the range on purpose, since there are clinical contexts, such as acute ischemia, in which [K^+^]_o_ can locally rise up to 9 mM or more [57].

The main effect of a decrease in [K^+^]_o_ should be a hyperpolarization of the cell membrane, due to a different Nernst potential for K^+^ ions. In addition, a [K^+^]_o_ decrease leads to a QT interval increase [28], a macroscopic marker of prolonged ventricular APD: therefore, a prolongation of atrial APD is expected as well [58]. On the contrary, ERP is expected to decrease [58, 59]: as experimentally observed by Downar et al. [60], APD and ERP may be “uncoupled” when varying [K^+^]_o_. Finally, while slowed cardiac tissue conductivity is a well-known effect of severe hyperkalemia, in the range of [K^+^]_o_ concentrations usually measured in HD patients, a positive dependence of conduction velocity on [K^+^]_o_ has been observed: this phenomenon is known as supernormal conduction [61–63]. Consistently, an increase in PWd during hemodialysis, significantly correlated to K^+^ decrease, has been reported [30]. In a previous simulation study [30] we have shown how both hypo- and hyperkalaemia can cause slowed cardiac tissue conductivity: in hypokalaemia, the RMP is significantly lower (hyperpolarized), and therefore the cell needs more time to reach the membrane potential threshold for AP upstroke; in hyperkalaemia, the RMP is significantly higher (depolarized) and, as a consequence, Na^+^ current availability is decreased and the current is much smaller than usual. In single cell simulations, a slow conduction can be associated to a smaller upstroke velocity and to an increase in the time needed for the voltage to rise toward the AP peak: AP_ud_ and *dV*/*dt*_MAX_ are then expected to show some kind of U-shape dependence when considering the full [K^+^]_o_ range.

**Figure 4:**
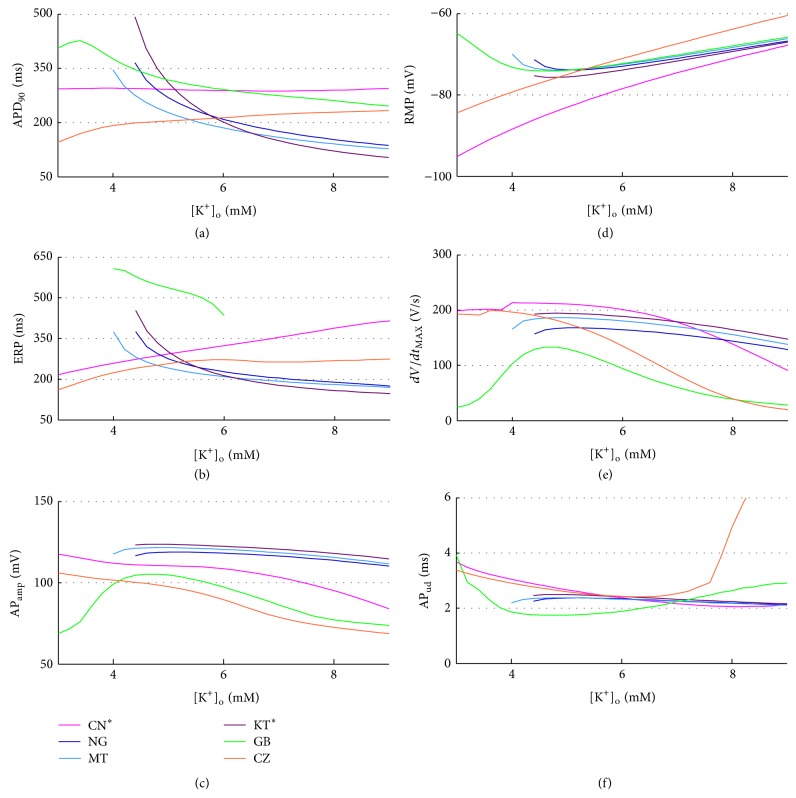
AP biomarkers versus [K^+^]_o_ for all the considered models.

A summary of the AP biomarkers for all the different [K^+^]_o_ is shown in Figure 4. When some models fail to repolarise with low [K^+^]_o_, the corresponding biomarkers have not been computed.

**Figure 5:**
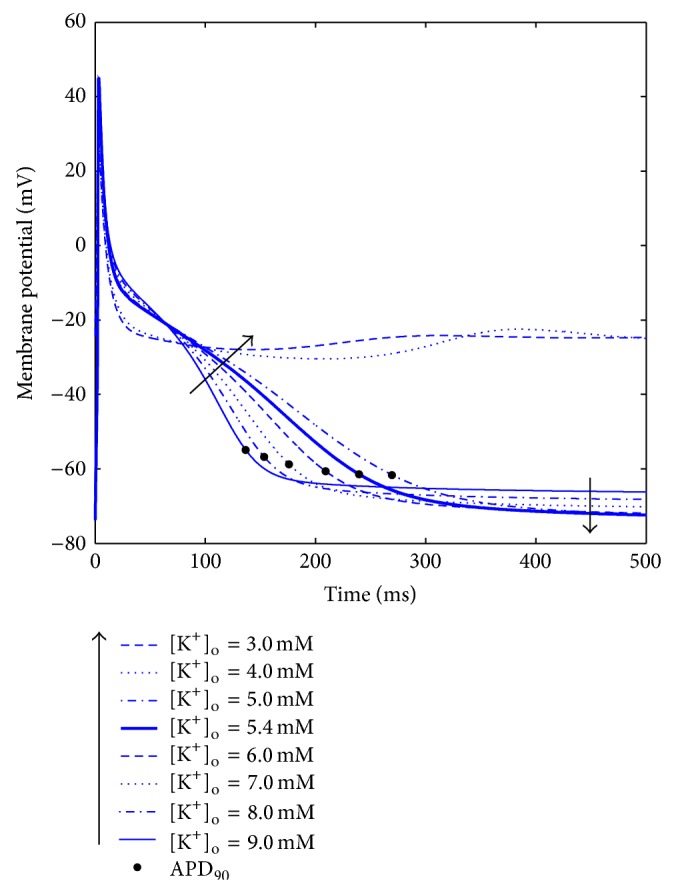
AP traces corresponding to different [K^+^]_o_ for the NG model. When decreasing [K^+^]_o_, the RMP becomes lower and the APD_90_ increases, both as expected. However, the model does not repolarise for [K^+^]_o_ values lower than 4 mM.

The models show quite different trends for some of the biomarkers, especially APD_90_ and ERP (Figures 4(a) and 4(b)). In NG, MT, and KT^*^ (Figure 4: blue, cyan, and purple traces), both the RMP and APD_90_ behave as expected; however, these models fail to repolarise when [K^+^]_o_ is set to low values, exhibiting early after depolarisations (EADs, see, e.g., Figure 5). Indeed, a decrease in [K^+^]_o_ leads to a reduction in the conductance of the K^+^ repolarising currents, that is, *I*_Kr_ and *I*_K1_, thus prolonging the APD: in these models this effect seems to be overdimensioned, probably due to a low repolarisation reserve, and therefore the membrane potential is not able to go back to its resting value. As an example, in Figure 5, shown are the AP traces corresponding to different [K^+^]_o_ levels for the NG model.

This is indeed a great limitation when aiming to apply these models to clinical contexts, since normal plasma K^+^ levels are between 3.5 and 5 mM and especially critical for HD patients because they need to remove the K^+^ accumulated during the interdialytic period, primarily in the intracellular pool, and therefore they usually end the HD session in hypokalaemia.

No significant changes have been observed in these models for *dV*/*dt*_MAX_ and AP_ud_, while the ERP follows the APD_90_ as expected. Finally, the AP_amp_ is inversely related to RMP.

As for the GB model (Figure 4: green traces), APD_90_ and RMP are quite similar to NG, MT, and KT^*^, but their trends change for low [K^+^]_o_: the model repolarises properly for all [K^+^]_o_, but when considering values lower than 4 mM the RMP is higher (more depolarised) than expected: therefore, APD_90_ and AP_amp_ are affected accordingly. AP_ud_ and *dV*/*dt*_MAX_ show the expected U-shape, related to a reduced conduction for both low and high [K^+^]_o_. As for the ERP, in this model it is always much longer than the corresponding APD_90_, even if it shows a similar dependence on [K^+^]_o_. In addition, probably due to the high stimulus amplitude needed to stimulate the GB model (as explained in the methods section), the ERP could not be computed for most of the [K^+^]_o_: when considering values below 4 mM or above 6 mM, the S_2_ AP peak was never lower than 80% of the corresponding S_1_, no matter how short the diastolic interval considered.

**Figure 6:**
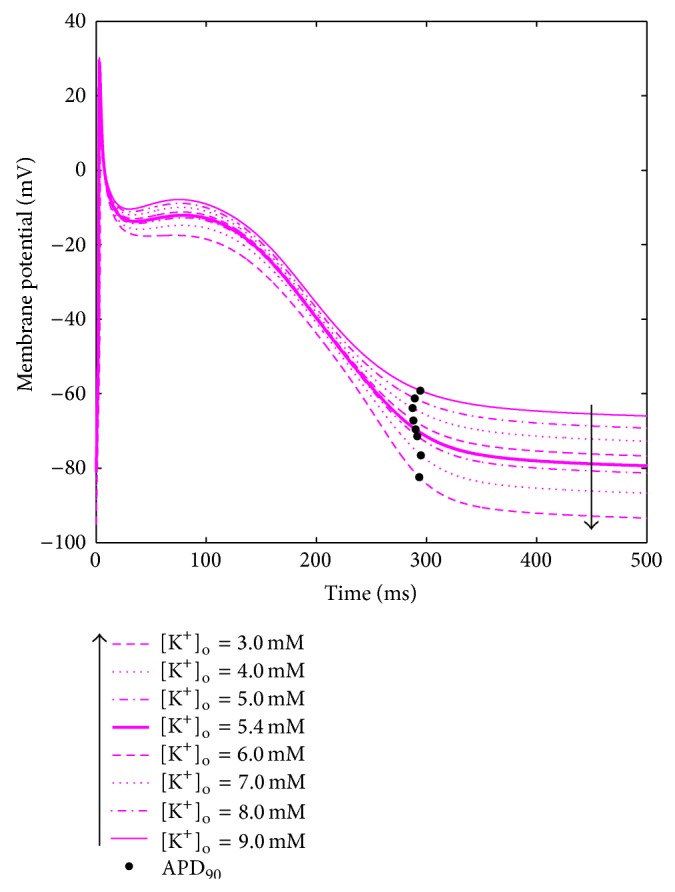
AP traces corresponding to different [K^+^]_o_ for the CN model. When decreasing [K^+^]_o_, the RMP becomes lower (as expected), but the APD_90_ is almost constant.

As for the CN^*^ and CZ models (Figure 4: pink and red lines), they develop a proper AP for all [K^+^]_o_ and they both show a very strong linear dependence of RMP on [K^+^]_o_: this dependence, by itself, should prolong the APD when decreasing [K^+^]_o_, since the membrane potential needs more time to repolarize and then reach its resting value. However, in these models the AP phase 2 shortens as well, so the overall APD_90_ is almost constant, or even decreasing with [K^+^]_o_, in contrast with the expected behaviour; this effect is even more pronounced when considering the ERP. As an example, in Figure 6 the AP traces corresponding to different [K^+^]_o_ levels for the CN^*^ model are shown.

In these two models, the AP_amp_ is again inversely related to RMP, and both AP_ud_ and *dV*/*dt*_MAX_ suggest a reduced conductivity in the [K^+^]_o_ range boundaries, especially when considering high [K^+^]_o_.

**Figure 7:**
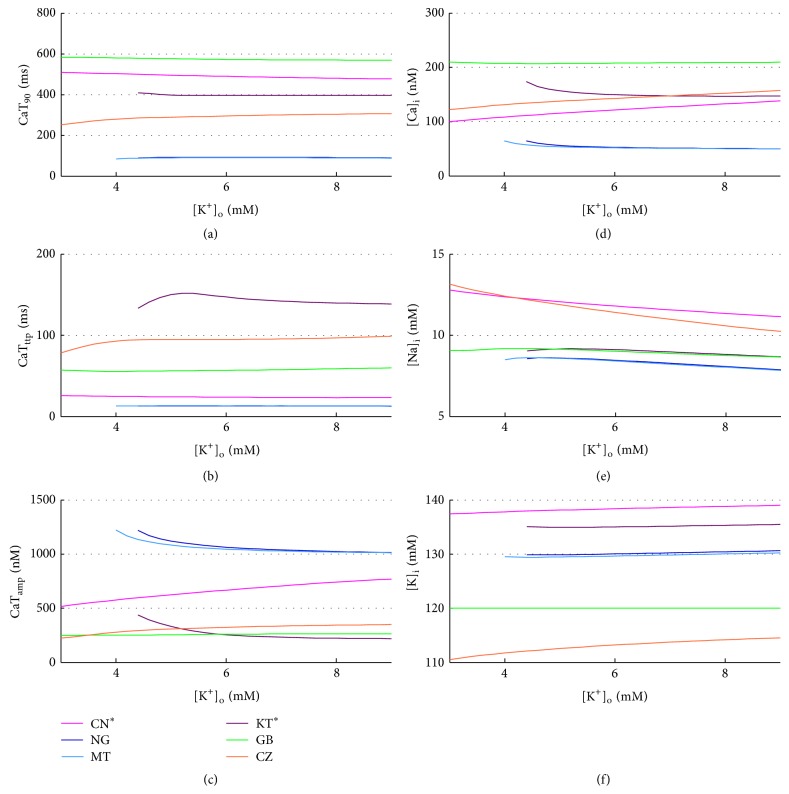
CaT biomarkers (left) and diastolic intracellular ionic concentrations (right) versus [K^+^]_o_ for all the considered atrial models.

No significant changes were found in CaT biomarkers and intracellular concentrations, in any of the considered models (Figure 7).

### 4.2. Calcium Variations

**Figure 8:**
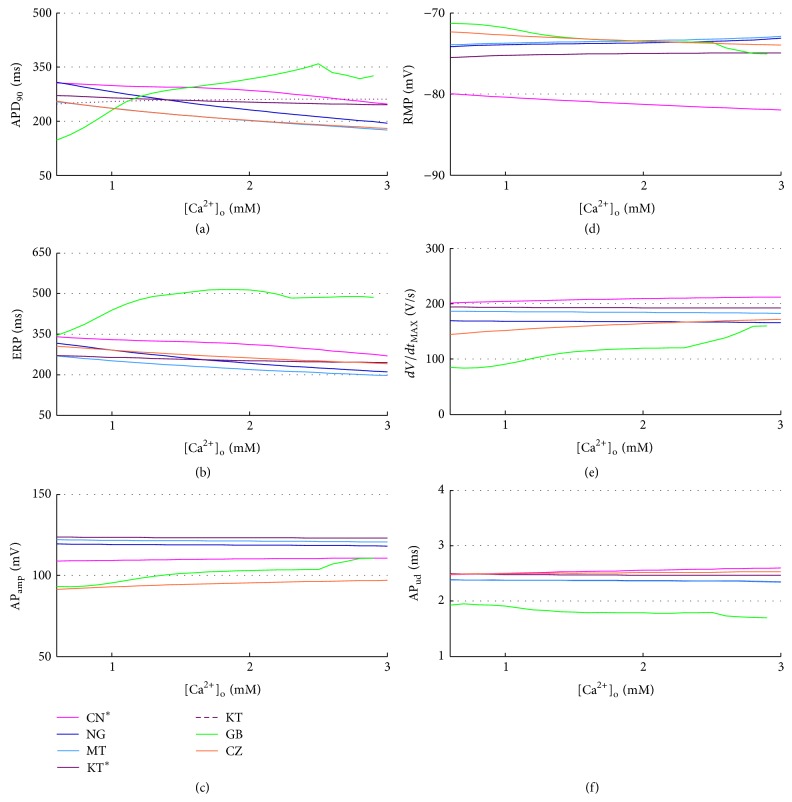
AP biomarkers versus [Ca^2+^]_o_ for all the considered models.

In all the models the extracellular Ca^2+^ concentration ([Ca^2+^]_o_) is set to the standard value used in the perfusion bath during V-clamp experiments, that is, 1.8 mM, which is quite high compared to the normal serum Ca^2+^ measured* in vivo* (1–1.3 mM), as discussed in detail in [64]. During a regular HD session, depending on the dialysis bath concentration, serum Ca^2+^ can either rise or decrease. Two previous simulation studies explored the effects of [Ca^2+^]_o_ on cardiac electrophysiology, considering the range 1–3 mM [64, 65]. However, serum Ca^2+^ is lower than 1 mM in several patients: reported pre-HD concentrations are, for example, 1.18 ± 0.09 mM in [30] and 1.06 ± 0.16 mM in [28]. Therefore, we decided to extend the explored range to 0.6–3 mM. A summary of the AP biomarkers for all the different [Ca^2+^]_o_ is shown in Figure 8.

The expected effect of [Ca^2+^]_o_ increase is a significant decrease of APD [64, 66]: the increment in driving force enhances the L-type Ca^2+^ current (*I*_CaL_) peak, but at the same time the Ca^2+^-dependent inactivation mechanism is strengthened, thus reducing the overall *I*_CaL_ and therefore shortening the APD. Even if the data showing this inverse relationship between APD and [Ca^2+^]_o_ has been recorded in ventricular cells, there are a few recordings confirming that the trend is the same for human atrial cells [64].

**Figure 9:**
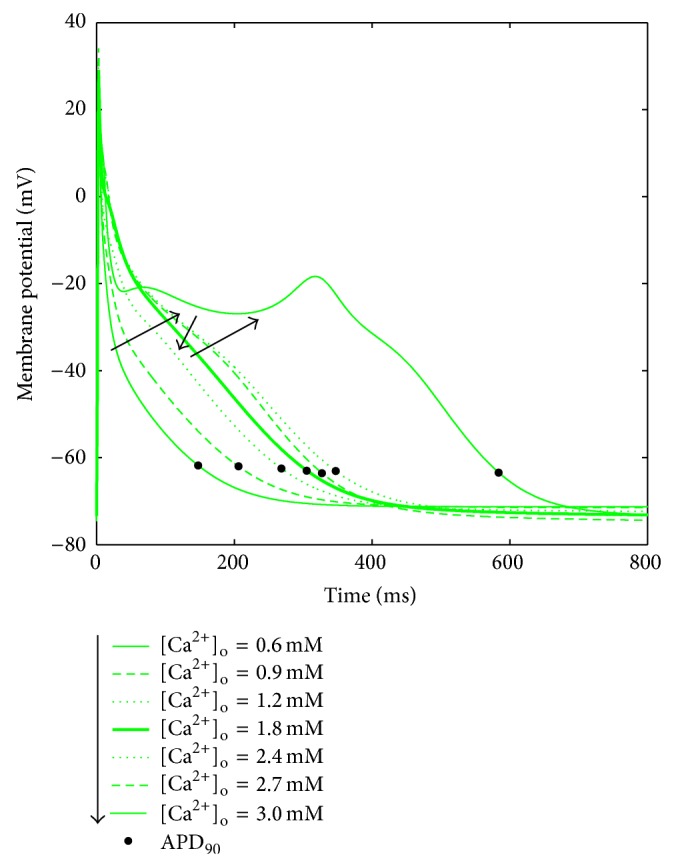
AP traces corresponding to different [Ca^2+^]_o_ for the GB model. When increasing [Ca^2+^]_o_, the APD_90_ first highly increases and then decreases. For [Ca^2+^]_o_ = 3 mM an EAD appears.

Indeed, CN, NG MT, KT^*^, and CZ models are able to reproduce this effect, together with a consistent reduction of ERP (Figures 8(a) and 8(b)). Notably, the original KT model shows an opposite trend for both APD_90_ and ERP, fixed in its improved version, where precisely the *I*_CaL_ formulation was changed. On the contrary, GB proves to be not very stable to [Ca^2+^]_o_ variations: APD_90_ and ERP show a biphasic trend, both considerably increasing with [Ca^2+^]_o_ from 0.6 to 2.5 mM and then decreasing until 3 mM, value in which EADs appear (Figure 9).

RMP, AP_amp_, and AP_ud_ are almost constant in all models. As for *dV*/*dt*_MAX_, only GB, CN, and CZ show a slight linear dependence with [Ca^2+^]_o_, related to the increase of *I*_CaL_ peak, which in these models has a greater contribution to the AP phase 0.

**Figure 10:**
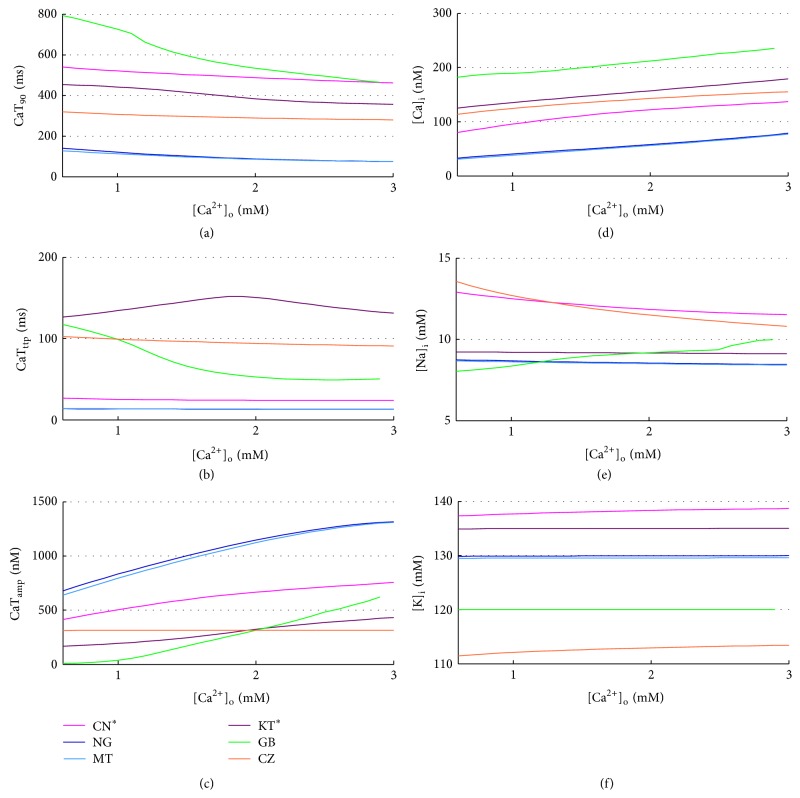
CaT biomarkers (left) and diastolic intracellular ionic concentrations (right) versus [Ca^2+^]_o_ for all the considered models.

As expected [66], diastolic Ca^2+^ increases with [Ca^2+^]_o_ for all the considered models (Figure 10(d)), whereas a couple of unexpected observations can be made on the CaT: CZ seems almost insensitive to [Ca^2+^]_o_ and GB fails to produce a significant CaT for [Ca^2+^]_o_ lower than 1 mM, in which CaT becomes really slow and almost negligible in amplitude. CaT_amp_ increases with [Ca^2+^]_o_ for all models (in agreement with [66]), while CaT timing (CaT_ttp_ e CaTD_90_) is not much affected by [Ca^2+^]_o_ (Figures 10(a), 10(b), and 10(c)) and neither is [K^+^]_*i*_ (Figure 10(f)).

On the contrary, [Na^+^]_*i*_ is finely tuned by Ca^2+^: the raise of [Ca^2+^]_o_ increases the outward Na^+^/Ca^2+^ exchanger current (*I*_NCX_), but the corresponding increase of intracellular Ca^2+^ contrasts this effect. At the same time, the Na^+^/K^+^ pump counteracts [Na^+^]_*i*_ variations, in both ways. As a result, [Na^+^]_*i*_ concentration is not much sensitive to [Ca^2+^]_o_ in most of the models (Figure 10(e)). However, in CN and CZ, the direct effect of [Ca^2+^]_o_ on the *I*_NCX_ plays the major role and as a consequence the [Na^+^]_*i*_ decreases slightly. In GB, instead, [Na^+^]_*i*_ increases, because when [Ca^2+^]_o_ increases the inward *I*_NCX_ is highly strengthened, due to the subsarcolemmal space in which [Ca^2+^]_*i*_ locally increases considerably.

Unfortunately, there are no experimental data available in literature on [Na^+^]_*i*_, *I*_NCX_, or Na^+^/K^+^ pump for different [Ca^2+^]_o_, either to confirm or to deny these findings.

### 4.3. Sodium and Volume Variations

In all the considered models the extracellular Na^+^ concentration ([Na^+^]_o_) is set to the standard value used in the perfusion bath during V-clamp experiments, that is, 130 mM for CN, GB, and CZ, 140 mM for NG, MT, and KT, in agreement with the normal serum levels of 135–145 mM. Na^+^ variation during a regular HD session is usually quite small (e.g., from 139.8 ± 3.4 to 141.6 ± 3.1 in [30], from 129/132 to 133/135 in [20]) and we explored the 120–150 mM range based on the corresponding data available in literature [67, 68].

**Figure 11:**
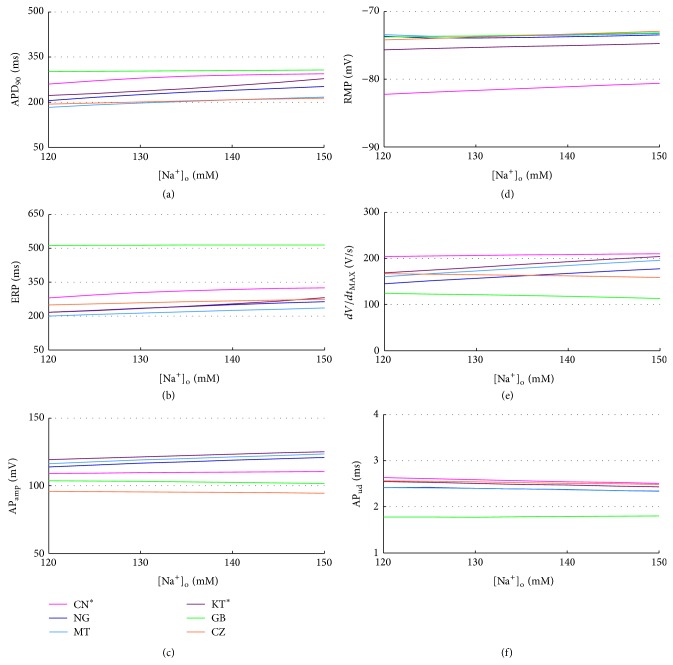
AP biomarkers versus [Na^+^]_o_ for all the considered models.

APD_90_ and ERP slightly increase with [Na^+^]_o_ in all the considered models except GB (Figures 11(a) and 11(b)), while RMP and AP_ud_ are almost constant (Figures 11(d) and 11(f)). In addition, in NG, MT, and KT *dV*/*dt*_MAX_ increase with [Na^+^]_o_, together with AP peak and therefore AP_amp_ (Figures 11(e) and 11(c)).

No significant differences were found in CaT biomarkers nor intracellular concentrations, apart from an increase in [Na^+^]_*i*_ (not shown). It is worth noting that, in CZ and CN, [Na^+^]_*i*_ regularly shows a stronger sensitivity to changes in extracellular concentrations.

**Figure 12:**
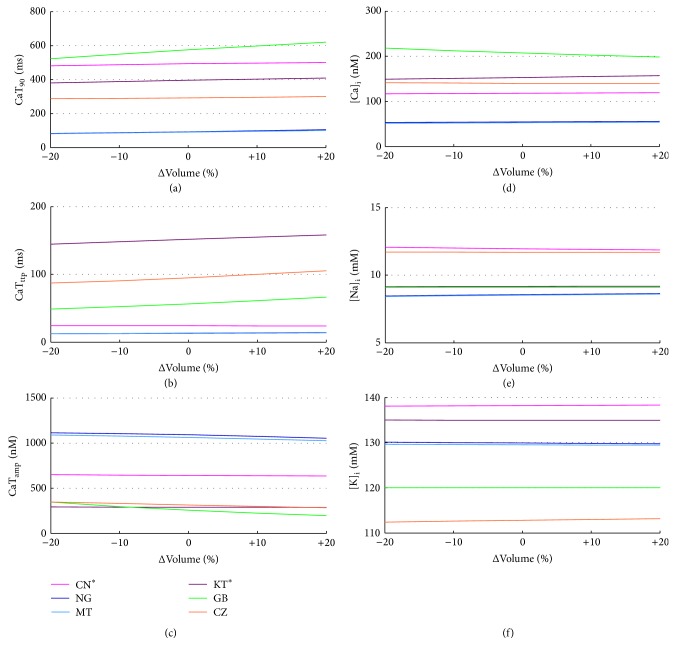
CaT biomarkers (left) and diastolic intracellular ionic concentrations (right) versus volume % changes for all the considered models.

Volume effects have been evaluated by scaling the intracellular volumes of ±20%. The corresponding AP biomarkers variations are all negligible (not shown); for example, KT shows the maximum APD_90_ change: +22.5 ms on the whole range. The CaTs become slightly slower when the volume increases, but notable changes have been found only in GB: CaTD_90_ increases of +104 ms on the whole volume range, together with an increase of CaT_ttp_ and a reduction of CaT_amp_ (Figures 12(a), 12(b), and 12(c)). In this model [Ca^2+^]_*i*_ also increases with volume, while no other significant changes occur in intracellular concentrations (Figures 12(d), 12(e), and 12(f)).

Unfortunately there are no experimental data on the effect of changes in [Na^+^]_o_ or volume on cardiac cells to either confirm or deny these findings.

### 4.4. Acetylcholine Effects

**Figure 13:**
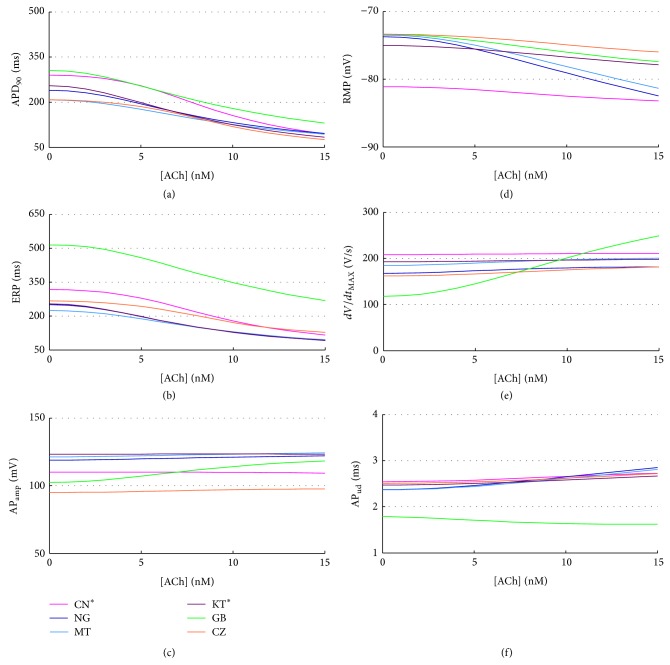
AP biomarkers versus ACh concentrations for all the considered models.

To analyse the effect of a possible increase in vagal activity, we simulated the effects of acetylcholine in the 0–15 nM range, adding to all models the same *I*_KACh_ formulation used in [5]. The expected effect of an additional outward K^+^ current is a more hyperpolarized RMP, together with a shortening of APD and ERP. This has been confirmed by experimental data [69–71] as well as by previous modeling studies [5, 72, 73]. When considering concentrations higher than 3 nM, all the considered models show a significant decrease of both APD_90_ and ERP (Figures 13(a) and 13(b)). In addition, the resting membrane potential is indeed hyperpolarised, especially in NG and MT (Figure 13(d)). The AP_ud_ is inversely related to RMP changes (Figure 13(f)), whereas AP_amp_ and *dV*/*dt*_MAX_ keep almost constant (Figures 13(c) and 13(e)); therefore, the overall conductivity is slowed down by ACh. In fact, starting from a more hyperpolarized potential with no significant changes in *dV*/*dt*_MAX_, the cell needs more time to reach the threshold for *I*_Na_ activation, to produce the upstroke. On the contrary, in GB both AP_amp_ and *dV*/*dt*_MAX_ increase with ACh, mostly due to a larger Na^+^ current for lower RMP, thus compensating this effect and limiting the theoretical AP_ud_ increase and the corresponding reduced conductivity.

Negligible effects were found in CaT biomarkers and intracellular concentrations (not shown).

## 5. Discussion and Conclusions

We have briefly pointed out that computational models of cardiac action potential (AP) have been successfully applied to investigate HD-related effects on the electrophysiology of different cardiac tissues (sinoatrial node, ventricle, and atrium) often leading to relevant interpretations of macroscopic observations made in clinical ECG and/or useful suggestions about HD treatment personalisation.

However, all these studies have been performed by using cardiac cell models that had been developed on the basis of* in vitro* experimental data, almost always acquired using standard Tyrode's solutions as extracellular fluid. It is obviously correct to simulate the electrical activity of cardiac cells by imposing the same conditions used in experimental protocols as far as the aim is a comparison with* in vitro* experimental data. On the contrary, it can be incorrect to use the same constant concentrations when the ultimate aim of simulations is the analysis of* in vivo*, dynamical conditions such as those during a HD session. Sometimes, this possible cause of discrepancy has been mitigated by few changes to the original models, for example, introduction of the effect of extracellular pH on the Na^+^/K^+^ pump activity in the DiFrancesco-Noble model of SAN cell [22] and strengthening of the *I*_CaL_ Ca^2+^-dependent inactivation in the Ten Tusscher-Panfilov model of human ventricular cell [28].

However, a systematic analysis of the applicability of cardiac cell models to reproduce the specific conditions occurring during HD or, in general, when the extracellular fluid composition changes is still lacking.

In the present paper, we addressed this kind of problem by focusing on human atrial cell models and on the following “cell environment” changes: extracellular electrolyte concentrations (K^+^, Ca^2+^, and Na^+^), cell volume, and acetylcholine.

We pointed out that several human atrial models are available, with significantly different behaviour upon such environment changes.

Unfortunately, experimental data on human atrial cells induced by extracellular concentrations changes are really rare in literature. This makes a stringent quantitative comparison between simulations and experimental measurements not possible for most of the considered electrophysiological properties. On the other hand, some qualitative behaviour is expected based on the overall evaluation of (i) knowledge of physiological mechanisms (e.g., the link between membrane resting potential and Nerst K^+^ potential); (ii)* in vitro* data measured in different cell types and species (e.g., [50, 64]); (iii)* in vivo* data on macroscopic ECG markers known to be related to atrial cellular electrophysiology (e.g., PWd).

We found a major problem in the NG, MT, and KT models: they all fail to repolarize and to produce physiological APs when [K^+^]_o_ is lower than 4 mM. This makes these models not appropriate to simulate the cardiac impact of HD. Indeed, the change in plasma [K^+^]_o_ is one of the more important and quantitatively large effects of HD, since K^+^ removal is one of the treatment aims and the end-HD [K^+^]_o_ is almost always much lower than 4 mM [30, 56]. Indeed, even in control condition ([K^+^]_o_ = 5.4 mM), the repolarising K^+^ currents (especially *I*_Kr_ and *I*_Ks_) of these models are quite tiny when compared to the ones of CN^*^ or CZ, who repolarise properly up to [K^+^]_o_ = 3 mM (current peaks are about 10 times smaller). In the NG paper the authors explicitly say that the *I*_Kr_ conductance has been reduced to fit AP data and that this current has been assigned a very low density [2]. Therefore, an increase of its conductance may improve the performance of these models for low [K^+^]_o_.

The GB model exhibits several shortcomings as well. First of all, although it produces a proper AP at all the tested [K^+^]_o_, it behaves nonphysiologically when [K^+^]_o_ is lower than 4 mM: the RMP depolarizes instead of hyperpolarize and, as a consequence, the APD_90_ also goes in the opposite way (shortening) and *dV*/*dt*_MAX_ dramatically decreases. Moreover, the excessive sensitivity to the amplitude of the stimulus current makes the computation of the ERP very unstable, leading to too long ERP values or no ERP at all. Since also in GB the repolarising K^+^ currents (both *I*_Kr_ and *I*_Ks_) are quite small in amplitude, increasing their magnitude may improve the model stability for low [K^+^]_o_ concentrations. Finally, the GB model responds poorly to [Ca^2+^]_o_ changes too: the APD_90_ trend is opposite to what is observed in human atrial cells [64]; that is, instead of showing an inverse dependence, it is increasing with [Ca^2+^]_o_, even displaying EADs for [Ca^2+^]_o_ equal to 3 mM, and the intracellular Ca^2+^ transient is almost nonexistent when [Ca^2+^]_o_ is lower than 1 mM. Therefore, the GB model turns out to be completely unsuitable to simulate the HD conditions.

As for the wrong dependency of APD on [Ca^2+^]_o_ a possible solution should address a modification of the L-type Ca^2+^ current, increasing the Ca^2+^-dependent inactivation with respect to the voltage-dependent one, in order to reduce the overall current for higher Ca^2+^ levels, despite the increase in driving force. Indeed, Ca^2+^-dependent inactivation seems to be underestimated in many AP models [64], and previous modeling works managed to reproduce the inverse APD-[Ca^2+^]_o_ just by strengthening this mechanism [65, 74].

The CN^*^ model responds properly to [K^+^]_o_ changes, at least from a qualitative point of view. It also reproduces well the “uncoupling” between APD and ERP variations when [K^+^]_o_ is increased (APD slightly decreases whereas ERP increases): this was experimentally reported by Downar et al. [60] when perfusing cardiac cells with hyperkalaemic “ischemic blood” and interpreted as a secondary effect to changes in resting potential, which is known to affect, in turn, the Na^+^ channels. In addition, simulation results for the CN^*^ model predict a decrease in intracellular Na^+^ when increasing [Ca^2+^]_o_: we are not aware of any available experimental data to confirm/deny this observation, which could have relevant implications.

The CZ model exhibits a good stability, with neither repolarization failure nor EADs occurrence. However, it also has a few discrepancies with respect to the expected behaviour: APD decreases with [K^+^]_o_, while the opposite should happen [28] and CaT_amp_ is insensitive to [Ca^2+^]_o_ while in all the other models it increases with it, in agreement with experimental data reported in [66].

As for the quantification of cardiac side-effects of HD therapy, overall simulation results confirm that changes in [K^+^]_o_ and [Ca^2+^]_o_ are the ones mostly affecting cellular electrophysiology [28, 30], whereas [Na^+^]_o_ and volume seem to have a minor impact. A qualitative summary of the expected variations in [K^+^]_o_ and [Ca^2+^]_o_ during HD and of the corresponding biomarker changes is shown in Tables 2 and 3, respectively, comparing experimental/computational data from the literature with the simulations results of this study.

Simulation results of acetylcholine effect show a reduction of APD and ERP in all the models, together with a more hyperpolarised RMP, in agreement with experimental data and previous modeling studies [5, 69–73]. In addition, all the models except GB show a reduction in AP_ud_, suggesting a slower conductivity, also consistent with the increased vulnerability to arrhythmias, such as AF, due to an increased vagal activity [20, 70]. However, there is no experimental evidence to confirm or deny these results, and a more detailed description of autonomic regulation should be considered for future improvements in computational modelling of acetylcholine effects.

Other HD-related effects (e.g., acidosis correction) have not been addressed in our analysis and are left to further investigations.

Finally, it is worth remembering that HD patients are first of all uremic patients: this pathological condition (e.g., “uremic intoxication”) can also affect some aspects of cardiac cellular electrophysiology and should be incorporated into the models. As a relevant example, downregulation of the Na^+^/K^+^ pump and high levels of circulating Na^+^ pump inhibitor have been reported in uremic patients compared to individuals with normal renal function, by several investigators [75–80].

In conclusion, computational modeling of human atrial cells constitutes a very useful tool to investigate the electrophysiological changes occurring in patients undergoing HD therapy. Nevertheless, it is always important to select carefully the specific model to use, depending on the particular aspect of interest. Currently, CN^*^ seems to be the more suitable human atrial model to analyse HD-related effects on atrial electrophysiology, though it is the oldest one and, therefore, it has a less detailed description of several cellular mechanisms.

Therefore, an additional model could be developed, trying to integrate and reconcile the knowledge of cellular and subcellular processes and their reactions to changes in the extracellular environment, taking into account the possible suggestions given above.

In this respect, work is still in progress in this specific field.

## Figures and Tables

**Table 1:** The human atrial AP models considered in this study and their main properties.

	CN/CN^*^	NG	MT	KT/KT^*^	GB	CZ
Cm (pF)	100	50	50	50	110	100
Cell volumes (pL)						
CYTO	13.67	5.88	5.88	8.10	21.45	13.67
SSL		0.12	0.12	—	0.66	—
JS		—	—	0.05	0.02	0.10
SR	1.21	0.44	0.44	0.18	1.16	0.27
Whole cell	20.10	49.42	49.42	13.90	33.01	20.10
I_stim_ (pA/pF)	15.0	19.6	19.2	18.8	19.5	12.0
# ODEs	21	29	30	43	62	39
Ref	[1]	[2]	[3]	[4, 14]	[5]	[6]
Year	1998	1998	2009	2011, 2014	2011	2013

Cm: membrane capacitance; CYTO: cytosol; SSL: subsarcolemmal space; JS: junctional space; SR: sarcoplasmic reticulum; I_stim_: stimulus current amplitude; # ODEs: number of differential equations in the model; Ref: reference paper; Year: year in which the paper was published.

**Table 2:** Qualitative summary of HD-induced [K^+^]_o_ variation on selected AP biomarkers.

[K^+^]_o_ ↘	RMP	APD	ERP	AP_ud_
	↘	*↗*	↘	*↗*
		[28, 60]	[59, 60]	[30, 59]
CN	−−	+	−−	++
NG	−	++	++, ∗	−, ∗
MT	−	++	++, ∗	−, ∗
KT^*^	−	++	++, ∗	−, ∗
GB	−	++	++, ∗	−, ∗
CZ	−−	−, ∗	− −	++

RMP: resting membrane potential; APD: AP duration; ERP: effective refractory period; APud: upstroke delay, inversely correlated with conduction velocity; black arrows: expected increase/decrease during HD, with the corresponding references, +/++/−/−−: moderate/large biomarker increase/decrease; ∗: biomarker changes different from the expected ones.

**Table 3:** Qualitative summary of HD-induced [Ca^2+^]_o_ variation on selected AP biomarkers. Results are shown for a Ca^2+^ increase, butdepending on the [Ca^2+^] concentration in the dialysis bath, a decrease could occur as well.

[Ca^2+^]_o_ *↗*	APD	ERP	CaTamp	[Ca^2+^]_i_	[Na^+^]_i_
	↘	↘	*↗*	*↗*	?
	[30, 64–66]		[66]	[66]
CN	−	−−	++	++	−
NG	−−	−−	++	++	=
MT	−−	−−	++	++	=
KT^*^	−	−−	+	++	=
GB	++, ∗	++, ∗	++	++	+
CZ	−−	−−	=, ∗	++	−

APD: AP duration; ERP: effective refractory period; CaTamp: Ca^2+^-transient amplitude; black arrows: expected increase/decrease during HD, with the corresponding references, +/++/−/−−: moderate/large biomarker increase/decrease; ∗: biomarker changes different from the expected ones.
